# Dyslexia and Attention Deficit Hyperactivity Disorder Associated to a De Novo 1p34.3 Microdeletion

**DOI:** 10.3390/genes13111926

**Published:** 2022-10-23

**Authors:** Ornella Galesi, Francesco Domenico Di Blasi, Lucia Grillo, Flaviana Elia, Maria Concetta Giambirtone, Maria Grazia Figura, Biagio Rizzo, Serafino Buono, Corrado Romano

**Affiliations:** 1 Laboratory of Medical Genetics, Oasi Research Institute-IRCCS, 94018 Troina, Italy; 2 Unit of Psychology, Oasi Research Institute-IRCCS, 94018 Troina, Italy; 3 Unit of Neurology and Clinical Neurophysiopathology, Oasi Research Institute-IRCCS, 94018 Troina, Italy; 4 Unit of Otorhinolaryngology, Oasi Research Institute-IRCCS, 94018 Troina, Italy; 5 Research Unit of Rare Diseases and Neurodevelopmental Disorders, Oasi Research Institute-IRCCS, 94018 Troina, Italy; 6 Department of Biomedical and Biotechnological Sciences, Medical Genetics, University of Catania, 95123 Catania, Italy

**Keywords:** dyslexia, attention deficit hyperactivity disorder, 1p34.3 microdeletion, *KIAA0319L* gene, *AGO1* gene

## Abstract

The authors report on a boy with dyslexia and attention deficit hyperactivity disorder. A protocol of standardized tests assessed the neuroadaptive profile, allowing deep neuropsychiatric phenotyping. In addition to the diagnosis of dyslexia and attention deficit hyperactivity disorder, such methodology led to endeavor cognitive, adaptive, and academic skills. Chromosomal microarray analysis detected a 452.4 Kb de novo heterozygous microdeletion in chromosomal region 1p34.3, including seven OMIM genes. The authors took a thorough evaluation of the association to the phenotype of the deleted genes. Further reports could strengthen such association.

## 1. Introduction

Dyslexia or reading disability (RD) consists in a deficit in reading ability in individuals with normal intelligence [1]. It is one of the most common neurodevelopmental disorders (NDDs), affecting about 5–12% of school-aged children [2]. RD is a multifactorial disorder with a strong genetic component and estimated heritability at 40–70% [3]. Some candidate genes have been implicated in RD, including *DYX1C1*, *CYP19A1*, *DCDC2*, *KIAA0319*, *ROBO1*, *C2Orf3*, and *MRPL19*, and some of these play a role in specific biological processes, such as migration of neurons during early brain development or out-growth of dendrites and axons [4,5,6]. Copy number variants (CNVs) contribute to language impairment in combination with the genetic background and the environment [7]. To date, very few studies in relatively small samples have assessed the contribution of CNVs to RD [6].

Attention deficit hyperactivity disorder (ADHD) is a disorder marked by pattern of inattention and/or hyperactivity-impulsivity, often persistent into later life and with a reported frequency in approximately 5% in school-aged children [8].

RD seems to co-exist most clearly with ADHD, and according to many studies, between 15 and 50% of individuals with ADHD have dyslexic problems and vice versa [9,10,11,12,13]. This association is genetically mediated [14].

Using chromosomal microarray analysis (CMA), we identified a single heterozygous microdeletion at 1p34.3 in a boy with RD and ADHD. The deleted interval, of approximately 452.4 Kb in size, is de novo and encompasses seven genes.

## 2. Case Report

The proband is the second-born boy of healthy, non-consanguineous parents. The paternal uncle shows paranoid schizophrenia and the paternal grandmother epilepsy, but no cognitive and genetic testing was performed.

His pregnancy was uneventful, and the delivery was at-term by cesarean section. At birth, weight was 2980 g (−1 SD), length 49 cm (+0.5 SD), and head circumference 34 cm (0 SD). Apgar scores were 10 at 1′ and 5′. He held his head up without support at 3–4 months, sat unsupported at 7–8 months, walked independently at 14 months, and spoke the first words at 18 months. At three years, he presented with hyperkinesis, oppositive behavior, and night awakenings (pavor nocturnus). He attends the fourth primary class at the current age of 9 years and 8 months. He is hyperactive and shows impairments in speech, language, and learning. Clinical genetics evaluation does not show any birth defect or morphologic anomaly.

## 3. Materials and Methods

### 3.1. Measures of Cognitive, Adaptive, and Academic Skills

A protocol of standardized tests assessed the neuroadaptive profile. The Raven’s Colored Progressive Matrices (CPM) evaluated nonverbal intelligence [15,16]; the Adaptive Behavior Assessment System—(second edition) (ABAS II) measured the adaptive behavior [17]; the Bell test revised (BTR) assessed the attention to details [18]; the Matching Familiar Figure test (MFFT) focused on the cognitive reflexivity-impulsivity style [19]; the Conners Parent Rating Scales—long version (CPRS-R:L) identified the presence of behavioral issues and ADHD [20]; the Achenbach Behavior Checklist (CBCL) measured behavioral issues [21]; the Peabody Picture Vocabulary Test for Italian-speaking subjects (PPVT-R) assessed receptive language [22]; the test for the reception of Grammar—2 (TROG-2) investigated the morphosyntactic comprehension skills [23]. Letters, words, and non-words lists and text reading investigated the reading abilities [24,25]. The spelling-to-dictation test assessed the spelling abilities [26].

### 3.2. Chromosomal Microarray Analysis

Whole-genome CMA analysis was performed on DNA isolated from peripheral blood lymphocytes drawn from the proband and his parents, using the standard Agilent SurePrint G3 Human CGH + SNP 4 × 180 K Microarray (Agilent Technologies, Santa Clara, CA, USA), with overall median probe spacing of 25.3 Kb. Labeling and hybridization were carried out according to the manufacturer’s instructions (Agilent Technologies, Santa Clara, CA, USA). The image of the array was acquired using the Agilent laser scanner G5761A (Agilent Technologies, Santa Clara, CA, USA) and analyzed with Agilent Cytogenomics software (v.5.0.2.5). Genomic coordinates were reported according to the GRCh37/hg19 genome assembly, and the data were compared to known copy number variation listed in available public databases, such as the *Database of Genomic Variants* (DGV, http://projects.tcag.ca/cgi-bin/variation/gbrowse/hg19, accessed on 31 August 2022), *DECIPHER* (http://decipher.sanger.ac.uk, accessed on 31 August 2022), and the *ClinVar* database (https://www.ncbi.nlm.nih.gov/clinvar, accessed on 31 August 2022).

No further genetic testing was performed.

## 4. Results

### 4.1. Cognitive, Adaptive, and Academic Skills

The administration of tests and multi-evaluation checklists led to the following cognitive, behavioral, and adaptive profiles. The Raven’s test was normal (38th percentile). The selective and sustained visuo-spatial attention function (BTR) fell below the 10th percentile; inattention and impulsiveness were confirmed by the MFFT test, with a score falling below the 5th percentile. The behavioral assessment forms (CPRS-R:L), completed by the caregiver, highlighted hyperactivity and inattention components: ADHD (95th–98th percentile), inattention (>98th percentile), and restlessness/impulsivity (95th–98th percentile), thus confirming significant behavioral issues. The behavioral assessment using the CBCL forms showed the presence of externalization problems (T score 70) falling within the clinical attention scope, while internalization problems (T score 60) were in the borderline scope. The subscales falling within the clinical range were those relating to attention (T score 70) and social (T score > 60) issues. Scores obtained at the ABAS-II relating to socio-adaptive skills were low. In the conceptual (CON 56), social (SO 58), and practical domains (PR 60), the scores were <2 SD. In addition to attention, these low scores might be due to the motor and behavioral impairments and dysfunctional educational styles within the boy’s daily life. The receptive lexicon (PPVT-R) was normal, and the receptive syntax (TROG-2) was below the normal range (<5th percentile). Decoding skills of letters, words, pseudo-words, and text were impaired for reading speed (<2 SD) and accuracy (<5th percentile). The administration of a text dictation unveiled a significantly deficient performance (<5th percentile).

### 4.2. Chromosomal Microarray Analysis

**Figure 1:**
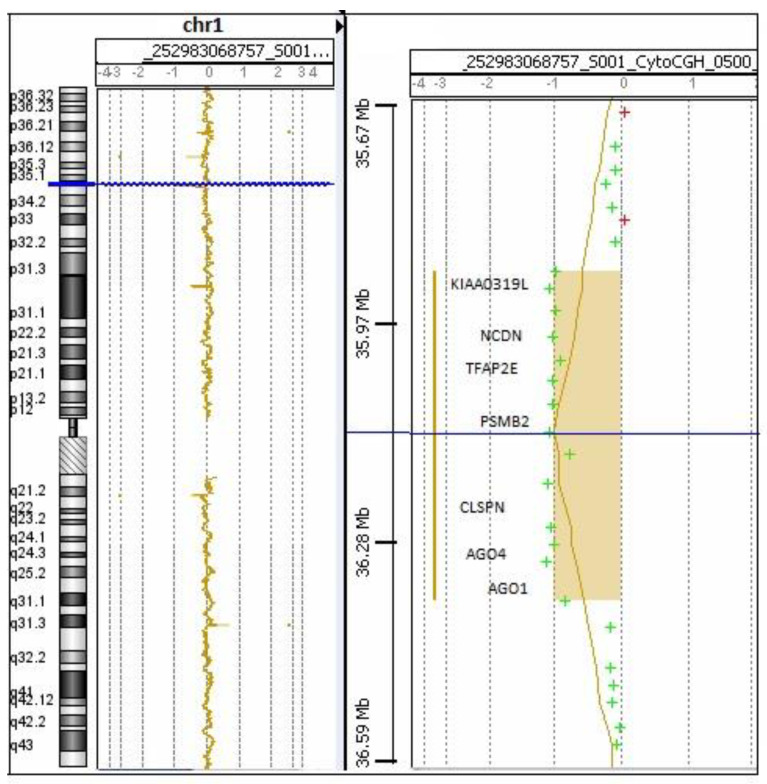
Array-CGH profile of chromosome 1 of our patient.

CMA displayed (Figure 1) a de novo 452.4 Kb heterozygous microdeletion in chromosomal region 1p34.3, ranging from 35,912,039 to 36,364,474 base pairs (ISCN 2020: arr [GRCh37] 1p34.3 (35,871,576 × 2, 35,912,039_36,364,474 × 1, 36,400,938 × 2). No other CNV was detected in the referred sample. Using the UCSC Genome Browser (http://genome.ucsc.edu/cgi-bin/hgGateway, accessed on 31 August 2022) and the *OMIM* database (http://www.ncbi.nlm.nih.gov/omim, accessed on 31 August 2022), we observed that the deleted region included the following OMIM genes: *KIAA0319L* (OMIM: 613535), *NCDN* (OMIM: 608458), *TFAP2E* (OMIM: 614428), *PSMB2* (OMIM: 602175), *CLSPN* (OMIM: 605434), *AGO4* (OMIM: 607356), and *AGO1* (OMIM: 606228). Breakpoint analysis revealed that the deletion includes the exons 1–16 of *KIAA0319L* (NM_024874.5) and the exons 1–8 of *AGO1* (NM_012199.5). A review of the DGV (Build GRCh37) revealed that there were no CNV in the general population similar in size and genomic endpoints to our patient’s deletion. The *DECIPHER* database yielded regions considerably larger than the deletion reported here. In the *ClinVar* database, a similar deletion was reported (VCV001526873.1). SNP analysis detected no region of copy neutral loss of heterozygosity in the proband. The patient is listed in the *DECIPHER* database as patient ID# 366790.

## 5. Discussion

This case report describes the detailed neuroadaptive profile of a patient with a de novo 452.4 Kb microdeletion in 1p34.3. He presented with a complex NDD, including severe RD, spelling disorder, speech delay, and ADHD. RD and its comorbidities, such as ADHD, are critical for educational performance, impairing adulthood. It is important to identify the etiology of reading and language disorders and their comorbidities, such as ADHD, to plan intervention strategies. The view that RD is caused largely by genetic factors is now generally accepted although the underlying etiology appears polygenic and multifactorial [4]. The microdeletion revealed in our patient includes the 5’ untranslated region (UTR) and the first 16 of 21 exons of the gene *KIAA0319L*, possibly leading to its haploinsufficiency. According to the *gnomAD* (https://gnomad.broadinstitute.org, accessed on 31 August 2022), the *KIAA0319L* gene has a probability of loss-of-function intolerance (pLI) of 0, and thus, heterozygous loss is less likely to be contributing to our patient’s clinical phenotype. However, the hypothesis that *KIAA0319L* is a candidate gene for RD is supported by suggestive linkage studies [27,28,29,30]. This is reinforced by its homology with another gene, *KIAA0319*, already associated to RD [31,32,33,34]. According to an NCBI protein-to-protein blast, both KIAA genes are 61% alike, with only 6% of the coding sequence lying in gaps. *KIAA0319L*, in adult mice, is expressed in brain regions that in the human brain are crucial for reading performance, supporting its possible involvement in RD [35]. KIAA0319L protein has physical interactions with Nogo Receptor 1 (NgR1), an axon guidance receptor. These two proteins interact predominantly in the cytoplasmic granules of cortical neurons in the human brain cortex. It can be inferred that KIAA0319L protein participates in axon guidance within the cerebral cortex [36]. Embryonic disruption of *KIAA0319L*, during rat corticogenesis, caused aberrant neuronal migration patterns with periventricular heterotopias [37]. Several reports highlighted how the impaired expression of other RD candidate genes, such as *KIAA0319*, *DCDC2*, and *ROBO1*, resulted in neuronal migration disorders [38,39,40]. This leads to hypothesizing the dysregulation of neuronal migration as the underpinning biological mechanism of RD. Observations made from post-mortem histopathological examination of dyslexic brains reporting anatomical abnormalities, such as cortical ectopias, heterotopias, and cortical dysplasia, reinforce the formulation of the hypothesis that RD is a neuronal migration disorder [41,42,43]. All these data support a contribution of the disruption of the *KIAA0319L* gene in the RD of our patient. The identification of *KIAA0319L* in a group of ADHD-associated genes and pathways suggests that the alteration of transcription, resulting from the deletion of its critical region, could also contribute to the complex NDD of our subject [44]. The *NCDN* gene encodes neurochondrin (NCDN), a cytoplasmatic neural protein of importance for neural growth, glutamate receptor signaling, and synaptic plasticity. The *gnomAD* database revealed that the *NCDN* is predicted to be severely intolerant to haploinsufficiency with a pLI score of 1. Conditional loss of *NCDN* in mice neural tissue causes depressive-like behaviors, impaired spatial learning, and epileptic seizures [45,46]. Monoallelic and biallelic variants in *NCDN* have been reported in six affected individuals with variable degrees of developmental delay (DD), intellectual disability (ID), and epilepsy [47]. Furthermore, heterozygous de novo deletions spanning 1.1 Mb to 3.1 Mb involving *NCDN* were identified in three individuals with ID and motor and speech delay but not seizures [48]. DD, ID, or epilepsy were absent in our patient who showed impairments in speech, language, and learning, suggesting that *NCDN* haploinsufficiency was associated to variable clinical features, therefore warranting further investigations for a more precise explanation. *TFAP2E* is a member of the AP2 family of transcription factors; such proteins bind to and regulate the promoters and enhancers of numerous genes involved in a wide spectrum of physiologic processes during development, cell cycle, and differentiation. *PSMB2* encodes a member of the B-type proteasome family, responsible for degradation of short-lived and misfolded cytosolic and nuclear proteins. *CLSPN* encodes an essential upstream regulator of checkpoint kinase 1 and triggers a checkpoint arrest of the cell cycle in response to replicative stress or DNA damage. This protein is also required for efficient DNA replication during a normal S phase. According to the *gnomAD* database (31 August 2022), *TFAP2E*, *PSMB2*, and *CLSPN* genes have a pLI score of 0, 0.91, and 0, respectively. No disruption of them has been implicated in the pathogenesis of RD and ADHD.

The deleted region also encompasses the *AGO4* gene and the first 8 of 19 exons of *AGO1* gene. AGO genes encode members of the argonaut family of proteins, which associate with small RNAs and have important roles in RNA interference and RNA silencing. According to the *gnomAD* database, the *AGO4* gene has a pLI score of 1, but to our knowledge, the effect in humans of *AGO4* dosage alterations has not been reported. Therefore, there is no direct evidence of a link between *AGO4* deletion and the phenotype in our patient. The pLi score of *AGO1*, provided in the *gnomAD* database, is 1, suggesting that haploinsufficiency is likely to be the main disease driver. De novo missense variants have been reported in *AGO1* in individuals with a broad spectrum of NDDs, including global DD, ID, autism spectrum disorder (ASD), hypotonia, dysmorphism, behavioral features, and language impairment with or without epilepsy [49,50,51,52]. The reported variants of *AGO1* gene are mainly nucleotide changes, while in our patient, a deletion including the first eight exons of the transcript was revealed. Large deletions at the 1p34.3 locus including *AGO1* together with *AGO3* (and sometimes *AGO4*), among other genes, were previously reported in five children with psychomotor DD as well as additional non-specific features, such as feeding difficulty, language impairment, and dysmorphic features [48]. These data support *AGO1* as a promising candidate gene for NDDs. This can be confirmed by the complex NDD including severe RD, spelling disorder, and ADHD of our case report. More variants and functional studies are necessary to reveal the real pathogenic mechanism of *AGO1*. Furthermore, a 2.6 Mb microdeletion in 1p34.3 involving the region of the present case was reported in a girl with severe DD predominantly affecting her language and fine motor skills [53]. ASD, abnormality of the outer ear, and global DD were reported through the *ClinVar* database in a patient with a similar deletion of uncertain significance. It would be interesting to know if RD was excluded or simply not reported. Table 1 lists a comparison of our case with some reported overlapping deletions.

Our case is the only one presenting with RD and not with DD or ID. That can be linked to the small size of deletion of our patient versus the many deleted genes in the other reports, resulting in a more severe phenotype hiding RD. In conclusion, we document a boy with RD and ADHD without major dysmorphism and ID/ASD, maintaining the likely role of genes included in the deletion. There is no real evidence that the ADHD and RD in our patient is due to the his CNV, as there are many cases reported where even larger euchromatic genes containing CNVs were found without any clinical consequences. The fact that a similar microdeletion was seen in *ClinVar* in an individual with a NDD is not sufficient to proof causation. There is no functional evidence that the CNV is the culprit of the disorder of our patient, and it could be a coincidence. The actual genetic cause of RD and ADHD in our patient remains to be fully elucidated. We encourage the report of individuals, whether patients or healthy, harboring overlapping microdeletions to investigate the clinical relevance and delineate the potential contribution to RD.

## Figures and Tables

**Table 1:** Comparison of our case with other overlapping deletions.

Reference	Age	Sex	Overlapping Genes	Deletion Coordinates 1p34.3 (hg19)	Phenotype
[53]	8 years	Female	*KIAA0319L*, *NCDN*, *TFAP2E*, *PSMB2, CLSPN*, *AGO4, AGO1*	34,859,671–37,468,932	Severe developmental delay, attention span/concentration deficit
[48] Proband 1	3 years 9 months	Female	*AGO1*	36,358,320–39,088,512	Developmental delay, dysmorphic features
[48] Proband 2	10 years 6 months	Female	*CLSPN*, *AGO4*, *AGO1*	36,154,687–38,591,548	Developmental and learning delays, dysmorphic features
[48] Proband 3	18 years	Female	*KIAA0319L*, *NCDN*, *TFAP2E*, *PSMB2*, *CLSPN*, *AGO4*, *AGO1*	35,933,018–37,052,682	Moderate ID, motor and speech delay, limited attention span, dysmorphic features
[48] Proband 4	17 months	Male	*KIAA0319L*, *NCDN*, *TFAP2E*, *PSMB2*, *CLSPN*, *AGO4*, *AGO1*	35,771,597–38,887,351	Motor and speech delay, dysmorphic features
[48] Proband 5	13 years	Male	*KIAA0319L*, *NCDN*, *TFAP2E*, *PSMB2*, *CLSPN*, *AGO4*, *AGO1*	35,447,244–36,643,150	Moderate intellectual disability, speech delay, hyperactivity and impulsivity, dysmorphic features
*ClinVar* VCV001526873	Not reported	Not reported	*KIAA0319L*, *NCDN*, *TFAP2E*, *PSMB2*, *CLSPN*, *AGO4*, *AGO1*	35,950,860–36,465,764	Autistic disorder, global developmental delay
Our case	9 years 8 months	Male	*KIAA0319L*, *NCDN*, *TFAP2E*, *PSMB2*, *CLSPN*, *AGO4*, *AGO1*	35,912,039–36,364,474	Dyslexia, attention deficit hyperactivity disorder

## Data Availability

Not applicable.
